# 3D Cell Culture in a Self-Assembled Nanofiber Environment

**DOI:** 10.1371/journal.pone.0162853

**Published:** 2016-09-15

**Authors:** Yi Wen Chai, Eu Han Lee, John D. Gubbe, John H. Brekke

**Affiliations:** BRTI Life Sciences, Two Harbors, MN, United States of America; Kyoto Daigaku, JAPAN

## Abstract

The development and utilization of three-dimensional cell culture platforms has been gaining more traction. Three-dimensional culture platforms are capable of mimicking *in vivo* microenvironments, which provide greater physiological relevance in comparison to conventional two-dimensional cultures. The majority of three-dimensional culture platforms are challenged by the lack of cell attachment, long polymerization times, and inclusion of undefined xenobiotics, and cytotoxic cross-linkers. In this study, we review the use of a highly defined material composed of naturally occurring compounds, hyaluronic acid and chitosan, known as Cell-Mate3D^TM^. Moreover, we provide an original measurement of Young’s modulus using a uniaxial unconfined compression method to elucidate the difference in microenvironment rigidity for acellular and cellular conditions. When hydrated into a tissue-like hybrid hydrocolloid/hydrogel, Cell-Mate3D^TM^ is a highly versatile three-dimensional culture platform that enables downstream applications such as flow cytometry, immunostaining, histological staining, and functional studies to be applied with relative ease.

## Introduction

Since their inception 130 years ago, two-dimensional (2D) cell culture methods have been instrumental in important discoveries in all disciplines of biological sciences, including genetics, cell biology, and tissue engineering. As these fields of study progress, the limitations of 2D cell culture are becoming evident as they fail to recapitulate the intricacies of biologic systems [1]. The shortcomings of 2D cell culture are further highlighted by studies showing that cell behaviors and gene expression are significantly influenced by the physical and mechanical properties of their microenvironments in three-dimensional (3D) [2–4]. 2D culture techniques have also been instrumental in the expansion of cancer biology discovery. Unfortunately, 95% of novel drug discoveries developed using 2D cell culture techniques fail to reach clinical practice [5,6]. The 2D culture drug discovery process essentially selects for a clonal population of cells from a tumor that can adapt to growing on a 2D, flat surface. As we understand, this adaptation leads to genetic drifts and alterations in gene expression. Therefore, 2D cultures are not effective cancer/tumor models [6,7] and is economically prohibitive.

Numerous 3D culture platforms including polymer-, protein-, and extracellular matrix (ECM) -based have been developed over the years; however, they each have limitations. Polymer and protein based materials can be cytotoxic and require long polymerizations times [8–14] while many ECM based materials are undefined and vary between batches. These batch variations have been known to affect reproducibility and unsuitable for clinical use [15]. Furthermore, these materials are not tissue-like, lack versatility, and can be difficult to handle. As the field of tissue engineering expands, demand is growing for new constructs composed of biologically “smart” materials; that is, materials capable of imparting mechanical and biochemical information to embedded cells [16–19]. Thus, a 3D, biomimetic, *in vitro* cell culture microenvironment is clearly needed within the fields of tissue engineering, regenerative medicine, and pharmacology [20–23].

The *in vitro* cell culture construct reviewed here, Cell-Mate3D™ (CM3D), is a 3D, malleable, microenvironment based on principles of cell and molecular biology. CM3D is a chemical “chimera” that integrates properties of an irreversible hydrocolloid with those of homogeneous hyaluronic acid (HA) and chitosan (CT) hydrogels. HA and CT are naturally occurring, linear polysaccharides with unique chemical properties [24–26]. HA is known as the first ECM of embryonic and early wound healing tissues [24,25] and a growing body of evidence describes CT’s biologic and antimicrobial properties [27–30]. When combined and hydrated into a hydrogel, CM3D becomes a tissue-like, versatile 3D culture platform. CM3D’s physical properties allow for nutrient and gas exchange, and cell migration, as well as polyelectrolytic (PEC) complex fibers that provide tissue-like structures for cellular interaction [31–35]. Hence, CM3D makes an attractive candidate for a 3D culture platform.

In this study, we demonstrate that CM3D brings additional dimensions to established methods such that we are able to image cells in their native 3D structure and their interactions with the surrounding matrix. Furthermore, we show that CM3D is highly versatile such that it can be decomposed to retrieve individual cells for flow cytometry analysis and functional migration assays.

## Materials & Methods

### CM3D Fabrication

The CM3D construct is fabricated by electrostatic interactions of its polysaccharide, constituent compounds: hyaluronan (HA-Na polyanion) and chitosan (CT-NH_3_^+^ polycation). These are originally presented as a blend of dry particles prepared according to a previously published protocol [36]. These dry particles adhere to one another by static electric, surface, charge (S1A Fig) and are blended at a mass ratio of CT-59%: HA-41% with a charge ratio (n^+^/n^-^) of 1.3. Its hydration fluid (HF) is composed of 10% LMD Dextran 40 in 5% Dextrose Injection USP (Dex-40), 0.9% Sodium Chloride Injection Solution USP, 0.6% glycerol phosphate solution (pH 6.5) and sterile water. This fluid is used to hydrate dry HA/CT particles at the fluid-to-dry mass ratio of 14μL/mg dry blend, as well as to re-suspend the pellet of cells destined for incorporation into CM3D’s microenvironment. The cell pellet is first incorporated into the hydration fluid by gentle mixing via a pipette, then deposited on Cell-Mate’s dry blend under continuous and gentle agitation to uniformly distribute the HF among the dry particles, reduce the fluid’s inherent surface tension on contact with the particles and deposit its cargo of cells uniformly throughout the construct’s volume. Immediately following hydration, HA and CT dry particles begin to dissolve in the HF solution and self-assemble to form insoluble, and irreversible, polyelectrolytic complex (PEC) fibers at the nanometer scale [37]. Over the next few minutes these fibers coalesce to form larger strands of insoluble PEC fibers at the micrometer scale (S1B Fig). Simultaneously, hydrogen bonds are generated within CT molecules as well as between CT and HA molecules [38–40]. This inter- and intramolecular hydrogen bonding provides CM3D with its initial mechanical properties and forms macromolecular networks in conjunction with developing PEC fibers that embrace/retain cells embedded with the HF. Suppliers of HA and CT possess biocompatibility data for each component (US FDA approvals and Drug Master Files). Combination of these two components, as described above, maintains the high levels of biocompatibility previously established for each at manufacture.

### Cell Culture in 2D

A human cervical cancer cell line (HeLa, ATCC®CCL-2) was purchased from the American Type Culture Collection (ATCC; Manassas, VA) and maintained according to standard mammalian tissue culture protocol and sterile technique. Cells were cultured in Eagle's Minimal Essential Medium (EMEM; ATCC®30–2003) and supplemented with 10% Fetal Bovine Serum (FBS, CAT#87–300, Hyclone Laboratories, Inc., USA) and 10U/mL Penicillin/Streptomycin (Cat#15140122, Thermo Fisher Scientific; Waltham, MA) at 37°C and 5% CO_2_. Cells were lifted with TrypLE (Cat#12604039, Thermo Fisher Scientific; Waltham, MA) and counted with the Muse Cell Analyzer (EMD Millipore; Billerca, MA)

### Cell Culture in CM3D

14 × 10^6^ HeLa cells were pelleted at 120g for 5 min, resuspended with HF and then mixed into the HA-CT dry blend by vigorous vortexing. The CM3D was formatted into a cylinder (S2 Fig) to yield a 0.5cm^3^ cell-loaded CM3D with ~14 × 10^6^ HeLa cells. The cylinder was cut into smaller 0.1cm^3^ cylindrical pieces for culture and other downstream applications. Each 0.1cm^3^ CM3D piece is cultured in a well of a 6-well plate with 6mL of HeLa culture medium/well. Culture medium was replaced every 3–4 days.

### Cell Viability

Cell viability within CM3D was measured using a LIVE/DEAD® Viability/Cytotoxicity Kit (Cat# R37601, Thermo Fisher Scientific; Waltham, MA), using the above-mentioned CM3D cell preparation. Cells were cultured for up to 28 days and viability was observed at various time points (Days 0, 1, 3, 7, 14, 21, and 28). The CM3D matrix was cut horizontally and incubated in 37°C and 5% CO_2_ for 15 minutes in 1mL of working Live/Dead stains. The CM3D was washed once in PBS for 5 minutes and immediately imaged with a Nikon A1 fluorescence inverted microscope (Nikon A1; Kanagawa, Japan) under the FITC and TRITC channels [41]. Cell viability was quantified by scoring for live (green) and dead (red) from five random fields of a large section and calculated using Eq 1. Images were analyzed with Fiji (Fiji is Just ImageJ; U. S. National Institutes of Health, Bethesda, MD) [42].

$$Cell\;Viability=\frac{Live\;Cell\;Count}{Live\;Cell\;Count+Dead\;Cell\;Count}\times 100\%$$ (1)

To help identify the efficacy of our stains on CM3D, we performed a negative control experiment on cells in CM3D that were killed with 70% ethanol for at least 15 minutes. The killed control cells serve two purposes in this experiment: 1) It allows us to adjust the settings on the microscope to minimize false negatives in the TRITC channel and to ensure that calcein AM and ethidium homodimer-1 are properly staining the cells in our matrix.

### Cell Proliferation

Cellular proliferation was assessed as a percentage of cells synthesizing DNA over time [43]. Approximately 2 x 10^6^ HeLa cells that were seeded in CM3D were labeled with 10μM 5-bromo-2-deoxyuridine (BrdU, Cat#B23151, Thermo Fisher Scientific; Waltham, MA) for 2 hours. CM3D samples were fixed in 10% Neutral Buffered Formalin for 3.5 hours at room temperature (RT) before it was paraffin embedded for sectioning. After the samples were sectioned, 3% hydrogen peroxide was used to block endogenous peroxide activity followed by heat retrieval. Immunofluorescence staining for BrdU was performed with the BD Pharmingen BrdU *In-Situ* Detection Kit (Cat#550803, BD Pharmingen; Franklin Lakes, NJ) by staining the sections in the biotinylated mouse anti-BrdU primary antibody. Immunofluorescence detection was achieved using a streptavidin Alexa Fluor 488 conjugate (Cat#32354, Thermo Fisher Scientific; Waltham, MA). Sections were counterstained with mounting media containing DAPI (Cat# F6057, Sigma-Aldrich; St. Louis, MO). Control stains were performed with positive and negative mouse intestine slides provided by the BD Pharmingen BrdU *In-Situ* Detection Kit. Slides were imaged with a Nikon A1 inverted microscope. BrdU incorporation was quantified in five, randomly selected, fields of a large 2 x 2mm section [44]. Cell proliferation (%) was calculated using Eq 2. Proliferating (green and blue) and non-proliferating cells (blue only) were quantified with FIJI.

$$Cell\;Proliferation=\frac{Proliferating\;Cells}{Proliferating\;Cells+Non\text{-}Proliferating\;Cells}\times 100\%$$ (2)

### Cell Retrieval from 3D

Cells embedded in CM3D were retrieved following the procedure outlined by BRTI Life Sciences (Cell Retrieval Kit, Cat#RET-1001, BRTI Life Sciences; Two Harbors, MN). Briefly, cells were retrieved from the CM3D matrix using 200uL of an enzyme blend consisting of 12.7μg/ul Chitosanase and 3.2μg/ul Hyaluronidase dissolved in a filter sterilized dilution buffer solution of 1mg/ml BSA in PBS. In a 24-well plate, a 0.1cm^3^ CM3D matrix piece was digested with 1mL of the sterilized enzyme blend solution for 15 minutes at 37°C in the CO_2_ incubator. In a biosafety cabinet, the CM3D matrix was vigorously broken up into smaller pieces using a 1ml pipet at every 3–4 minutes for 15 minutes. The disintegrated matrix was filtered out through a 100μm cell strainer (Miltenyi Biotec; Bergisch Gladbach, Germany) into a 50ml conical tube. The contents of the strainer were rinsed out with media using some agitation then centrifuged at 120 x g for 5 minutes to separate the supernatant from the pelleted cells.

### Flow Cytometry Analysis

Cells retrieved from CM3D were characterized using the following antibodies: CD44-Alexa Fluor 488 (Cat#103015, Biolegend; San Diego, CA) and CD24-Brilliant Violet 421 (Cat#101235, Biolegend; San Diego, CA). Retrieved cells (2 x 10^5^ cells) were incubated with antibodies in fluorescence-activated cell sorting (FACs) buffer (PBS, 1% BSA) for 30 minutes on ice, washed with PBS, fixed with 2% Paraformaldehyde. Cells were passed through a strainer prior to analysis on the LSR II Flow Cytometer (BD BioSciences; Franklin Lakes, NJ).

### Scanning Electron Microscopy

0.1cm^3^ sections of CM3D with HeLa cells were fixed in 3% paraformaldehyde and 1% glutaraldehyde in a 0.1M sodium cacodylate buffer (pH 7.4) with 2% sucrose, 5mM calcium chloride, 5mM magnesium chloride for 3.5 hours on an oscillating shaker. The specimens were gently rinsed in buffer for 10 minutes then placed in 1% osmium tetroxide and 0.1M sodium cacodylate buffer overnight before washed in ultrapure water (NANOpure Infinity®, Barnstead/Thermo Fisher Scientific; Waltham, Maryland). The specimens were then dehydrated through successive gradients of ethanol (25%, 50%, 70%, 95% and 100%). Once the specimens were in 100% ethanol, they were immersed in liquid nitrogen, placed on a brass surface submerged in liquid nitrogen, and fractured into smaller pieces using a pre-chilled wooden dowel. The pieces were then returned to 100% ethanol and processed in a critical point dryer (Autosamdri-814; Tousimis; Rockville, Maryland). The samples were mounted on aluminum stubs with double-sided carbon adhesive tabs, sputter coated with gold-palladium or platinum, and observed in a scanning electron microscope (S3500N, Hitachi High Technologies America, Inc.; Schaumberg, Illinois) at an accelerating voltage of 5.00kV.

### Immunofluorescence & Confocal Microscopy

HeLa cells were cultured in 2D monolayers or CM3D hydrogels for 3 days. For the CM3D preparation, a 0.25cm^3^ sized CM3D was loaded with 4 × 10^6^ cells and divided into four 0.05cm^3^ pieces. After 3 days of culture, the CM3D sections samples were fixed in 4% paraformaldehyde for 45 minutes and washed three times with PBS. Prior to staining, a 0.05cm^3^ sized cocoon was broken up into 1-2mm^3^ pieces and permeabilized with 0.5% Triton-X 100 in PBS for 10 minutes at 4°C. The samples were washed with PBS and blocked with 0.5% BSA, 0.5% Fish skin gelatin in PBS for 1 hour at room temperature with rocking. After blocking, cells were incubated with primary antibody (Vinculin, Cat#MAB3574-C, EMD Millipore; Damstadt, Germany) for an hour at room temperature with rocking. Cells were then incubated with secondary antibody (Anti-Mouse IgG1, Alexa Fluor 568, Cat#A21124, Thermo Fisher Scientific; Waltham, MA) and phalloidin, Alexa Fluor 488 (Cat#A12379, Thermo Fisher Scientific; Waltham, MA for 45 minutes at room temperature. After each antibody incubation, the samples were washed three times for 5 minutes each in 0.1% Tween 20 in. Two drops of NucBlue® Fixed Cell ReadyProbes® Reagent (Cat#R37606, Thermo Fisher Scientific; Waltham, MA) was used to stain cell nuclei for 5 minutes. All reactions were carried out in a 1.5mL micro centrifuge tube. Samples were immediately imaged on a Nikon A1 confocal microscope and images analyzed with the NIS Elements imaging software and FIJI.

### Mechanical Properties Testing of Acellular and Cellular CM3D

To assess the mechanical properties of our hydrogel, compression tests were performed to measure the elastic properties of our biomaterial with (cellular CM3D) and without cells (acellular CM3D). HeLa cells were seeded in a 0.1cm^3^ CM3D and cultured in parallel with acellular CM3D in culture medium at 37°C and 5% CO_2_ for 36 hours. Measurements were taken at various time points (1-, 6-, 12-, 24-, 30-, and 36 hours) and the mechanical properties of acellular and cellular CM3D were characterized on the Electroforce 5500 test instrument (TA Instruments, Eden Prairie, MN, USA). To simulate a natural physiological environment of cells, all compression tests were performed in a culture dish with PBS at room temperature. A cylindrical spacer of 0.1cm^3^ thickness diameter was made to place in between the compression platens to aid in setting a consistent gauge length for the measured CM3D sample. The mechanical properties (dynamic stiffness and elastic modulus) of the samples were characterized under 54% of maximum compression force with a constant compression rate of 0.01mm/sec. The Stress-Strain curve was calculated using the following equations [45]:

$$Stress=\left[\frac{Load\;(g_{f})}{Area\;({mm}^{2})}\right]\times \left[\frac{9.8N}{{kg}_{f}}\times \frac{1{kg}_{f}}{1000g_{f}}\right]\times \left[\frac{1000^{2}{mm}^{2}}{m^{2}}\right]$$ (3)

$$Strain=\frac{displacement\;(mm)}{gauge\;length\;(mm)}$$ (4)

### Migration

To assess CM3D functional assay performance, we selected to model the migratory ability of HeLa cells toward a chemoattractant over time. CM3D was seeded with cells and serum starved (1% FBS in EMEM media) for 1 day. Cultures were transferred to new plates containing chemoattractant, 20% Fetal Bovine Serum (FBS) for 1 day. Cells were harvested from the media and bottom of the plates (by trypsinization) and counted with the Muse cell analyzer (EMD Millipore; Damstadt, Germany). New plates were used between each media component change. The schematic illustration (S3 Fig) outlines the progression of steps for this study.

### Statistics

Statistical analysis was performed using GraphPad Prism version 7 (GraphPad Software, La Jolla, CA). For comparisons of more than two groups, the one-way ANOVA was used while the student t-test was performed between two groups. Due to the complex skewed nature of the data, non-parametric statistics were used throughout. Levels of significance are indicated accordingly: ns > 0.05, *p ≤ 0.05, **p ≤ 0.01, ***p ≤ 0.001 ****p ≤ 0.0001.

## Results

### Cell Viability and Proliferation in CM3D Construct

**Fig 1:**
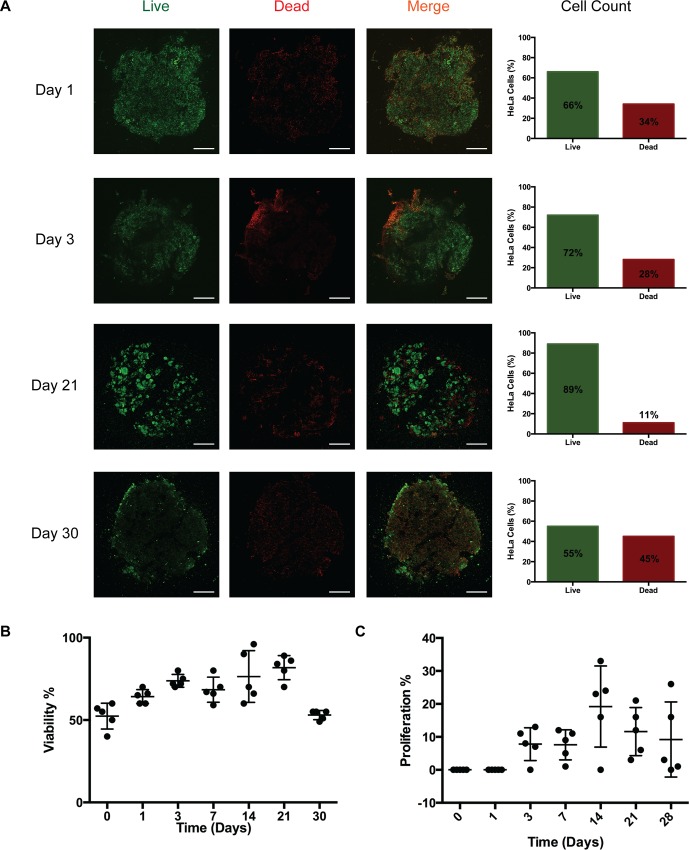
Viability over time in CM3D. A) HeLa CM3D cultured cells were stained with calcein AM (live-green) and EthD-1 homodimer (dead-red). Cross sections of the stained CM3D cultures were imaged at 4X magnification. Cell viability is represented in the bar graph as a mean percentage of live cells over total live and dead cells. Scale bar = 1000um. B) Scatter plot representing HeLa cell viability trend in CM3D over 30 days. SD error bars for each time point was analyzed from the live percentage in five fields of one experiment cross section (n = 1). C) The scatter plot represents BrdU incorporation into cells over time. BrdU positive cells were scored in 5 randomly selected fields from a cross section of a CM3D (n = 1) and is represented as a percentage of proliferating cells over total proliferating and non-proliferating cells. Results are expressed as a ±SD of the proliferation percentage over 28 days.

In order to evaluate cell viability in long-term CM3D cultures, we monitored cell viability with calcein AM and ethidium homodimer-1 staining. Our results demonstrate that cell viability increased from 66% on day 1 to 77% on day 3. Interestingly, the trend in cell viability rose between day 7 to day 21, from 67% to 89%, respectively. Towards the end of the experiment, cell viability sharply decreased between day 21 to day 30, from 89% to 55%, respectively (Fig 1A and 1B). This observation is consistent with other studies where viability is reduced in long-term 3D culture systems [46,47] due to the presence of oxygen and nutrient gradient [48].

Next, we wanted to evaluate if CM3D supports HeLa cell proliferation. Since CM3D cultures contain very high cell densities, we measured the proliferative capacity of HeLa cells in CM3D by BrdU incorporation into genomic DNA (31). As we expected, no proliferation was observed at the initial time points (day 0 and 1). However, proliferation resumed after 3 days of culture to 7% and plateaued at day 7 (Fig 1C). Between day 7 and 14 we noticed a sharp increase in cell proliferation, from 7% to 24%, respectively. In the final leg of the experiment, proliferation rates dropped from 14% at day 21 to 10% at day 28. In summary, CM3D supports long-term cell culture by maintaining cell viability and proliferative capacity.

### Cell Isolation and Flow Cytometry

One important feature of the CM3D culture system is that single cells can be retrieved from the hydrogel for downstream analysis. Through our cell retrieval process, we demonstrated a method to decompose the CM3D construct with hyaluronidase and chitosanase enzymes to retrieve cells. Since the enzymes may have non-specific activities, we were concerned about the preservation of cell surface markers after being exposed to the chitosanase and hyaluronidase enzymes. Cell phenotype was evaluated following staining and flow cytometry analysis for the following surface antigens; cancer stem cell markers, CD44 and CD24 [49].

**Fig 2:**
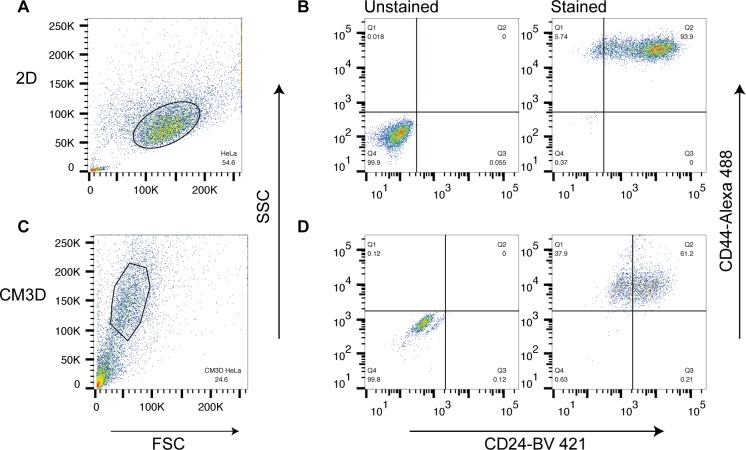
Flow Cytometry analysis of cells isolated from CM3D cultures vs 2D cultures. A) & C) SSC vs FSC plots from 2D cultures and cells isolated from CM3D cultures, respectively. Gates were set accordingly to exclude debris. B) & D) Cells were gated for CD44 (x-axis) and CD24 (y-axis).

Based on the forward and side scatter plots, we found that cells from 2D cultures were larger in comparison to cells retrieved from CM3D cultures (Fig 2A and 2C). This is consistent with previous studies showing that cells cultured in 3D experience morphological changes including cell size [50].

When analyzed for cell surface markers, cells from 2D cultures and CM3D cultures were positive for CD44+ and CD24+ markers (Fig 2B and 2D). The staining pattern of CD44 is present on all HeLa cells, whereas CD24 is unevenly expressed in cells (Fig 2B), which is in agreement with other studies [51]. This observation strongly demonstrates that the cell surface markers were left intact after the cell retrieval process from CM3D.

Unlike many 3D hydrogels, CM3D offers a safe and easy method to retrieve cells from a 3D culture system without affecting their cell surface markers. Although the staining pattern of from 3D cultures appear dimmer (Fig 2D), we believe this is due to the effects of 3D culture that alters global gene expression patterns instead of cell retrieval process.

### Analysis of Cell Matrix Interface with SEM

**Fig 3:**
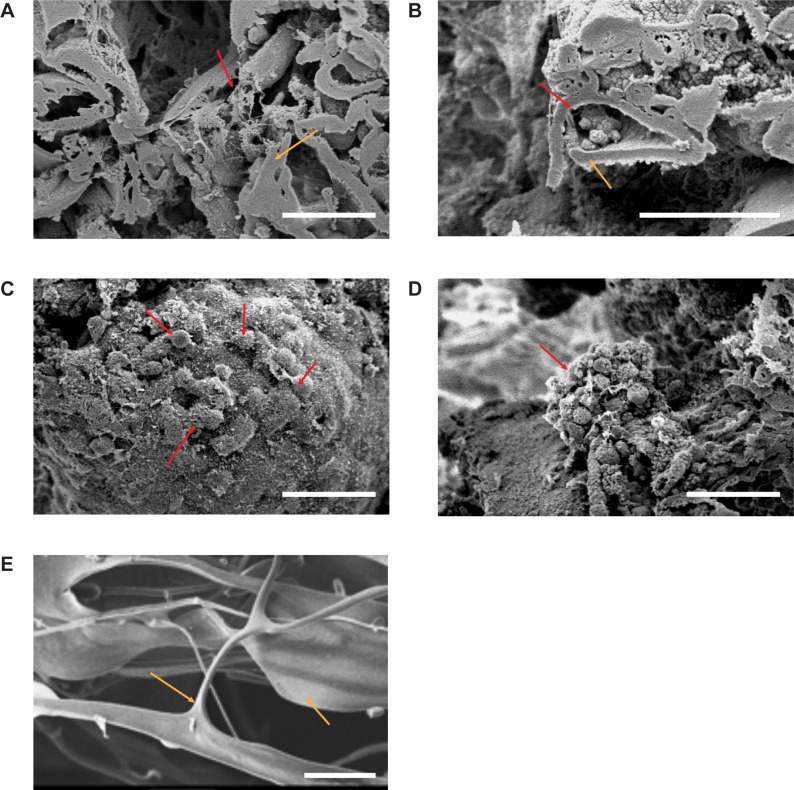
SEM images of acellular and cellular CM3D. A) Interior, cross sectional surfaces produced by freeze fracture of acellular CM3D, showing the interaction between HA (red arrow) and CT (orange arrow) on Day 21 (Original Magnification 700X). B) Interaction of cells with CM3D on Day 28. Cells within CM3D (red arrow) grow inside the crevices created by the formation of HA-CT fibers (Orig. Mag. 1000X). C) Interaction of cells and CM3D on day 21. Cells are spread out on the periphery (red arrow) of CM3D while some cells appear to be interacting with one another (700X). D) Cell to cell interaction as an aggregate cell mass (red arrow) attached to the periphery of CM3D (700X). E) CT only shard imaged prior to being blended with HA. This image was taken on its surface. The white scale bar for all images is 50μm in length.

Through SEM, we verified the interaction between CM3D’s natural components, CT and HA, after proper hydration with HF (Fig 3A). CT displays a homogeneous substance of monotonous topography while HA presents as a lace-like, diaphanous velour, when found independently and as a filamentous coating on chitosan surfaces (S1B Fig). The CT layers fuse with HA leaflets throughout the matrix to form pockets for cells to grow in (Fig 3B).

In our SEM analysis of cellular CM3D, we were able to observe cell adherence to CM3D on its surface and within the matrix after one day in culture (Fig 3C). Over time, cells were easier to locate and image on the periphery of CM3D. Viable cells were characterized by having a HA coat (S4A Fig) and an attachment to CM3D while non-viable cells looked perfectly spherical and unattached to the matrix surface (S4B Fig). The periphery of CM3D showed different types of cell morphology in a 3D system. There were areas that had layers of cells and other areas that had aggregates of cells (Fig 3D). Both morphological forms exhibit cell-matrix and cell-cell interaction. In order to distinguish CT from HA, a CT only image was taken by SEM to show its dense, smooth and featureless morphology (Fig 3E)[52].

### Cell Morphology in CM3D Construct

Unlike most other 3D-culture platforms [53,54] CM3D-cultures can be easily fixed and stained with conventional 2D-culture immunostaining methods. Immunostaining of CM3D-cultures allows the visualization of cells in their native 3D morphology in a 3D environment, otherwise not possible in 2D cultures. Next, we labeled cellular actin fibers with phalloidin and focal adhesion sites with Vinculin [55,56] in CM3D- and 2D-cultures.

**Fig 4:**
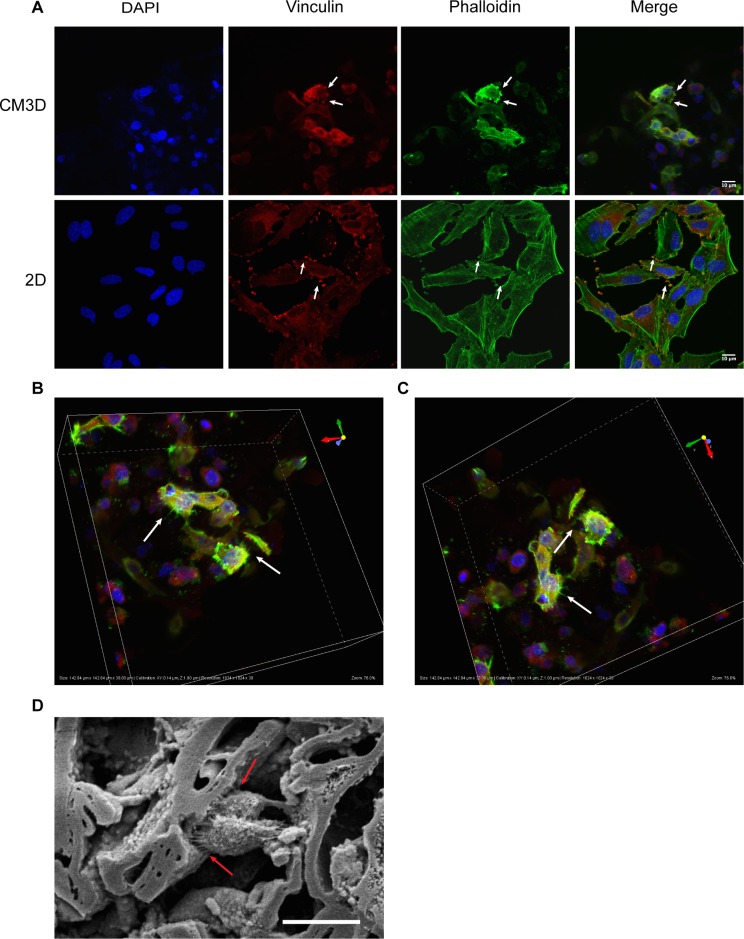
Fluorescence analysis of cells in CM3D. A) 60X confocal images of HeLa cells cultured in 2D or CM3D. DAPI (blue) was used for nuclear staining, Vinculin (red) was labeled with with Alexa Flour 568 (red), and phalloidin (green) for actin fibers. White arrows indicate focal adhesion sites. B) & C) 3D rendering of HeLa CM3D cultures. White arrows indicate lamellipodia. D) SEM image of cells interacting with the PEC fibers of CM3D. Black arrows indicate lamellipodia interacting and attaching to the surrounding matrix.

As expected, our results show striking differences in cellular morphology between the two types of cultures (Fig 4). Actin (phalloidin) staining shows the homogenous affect 2D cultures, such that the cells are flat, geometric, and spread out with relatively smooth edges (Fig 4A). In CM3D cultures, however, we observed various types of morphologies such as migrating cells that are polarized and irregularly shaped as well as resting cells in the background that appear rounded (Fig 4B). Additionally, we observed that cells cultured in 2D are larger in comparison to cells cultured in CM3D, which is consistent with our flow cytometry data (Fig 2) and other studies [50,57].

In 2D-cultures, focal adhesion complexes and actin fibers were observed only on the plane of the culture dish. However, in CM3D cultures we observed contrasting actin and vinculin staining patterns such that, focal adhesions were found to form on multiple planes of the matrix (Fig 4B and 4C). In CM3D cultures, we were able to image thick Z-section of 38 microns of this sample and found that cells were projecting very prominent lamellipodia that appear to be interacting with neighboring cells. We also noticed that these lamellipodia were interacting with the surrounding matrix (unstained) (Fig 4B); however, we could not confirm this through confocal microscopy due to a lack of matrix strain. We then imaged CM3D cultures through SEM and we were able to confirm that cells were indeed interacting and forming bridges with the surrounding matrix through SEM imaging (Fig 4D).

We believe CM3D is capable of providing more insights into studying cells in 3D, which are more physiologically relevant. With this novel material, we were able to bypass the limitations of imaging in 2D- and certain 3D-culture platforms. Evidently, from our results, we are able to image thick Z sections for analyzing how cells interact with each other and their microenvironment in all three spatial dimensions in their native 3D morphology with minimal manipulation.

### Mechanical Force/Stress Test of CM3D

**Fig 5:**
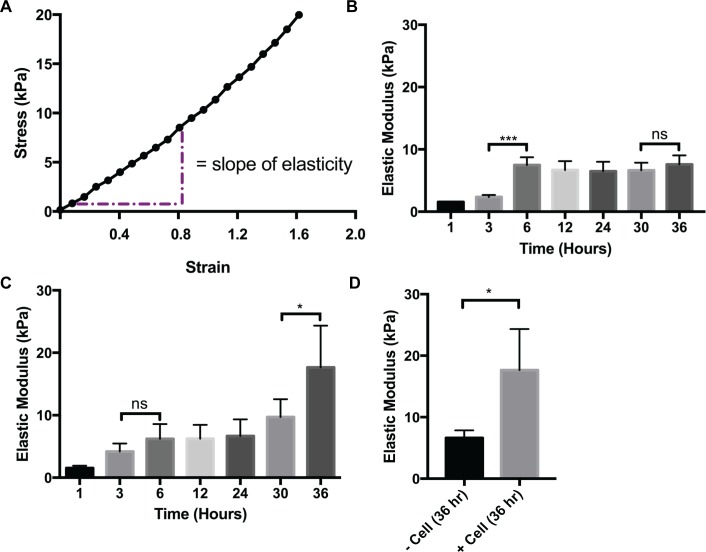
Mechanical Properties Analysis of CM3D. A) Region of elastic modulus for CM3D stiffness. B) Graph representing acellular (- cells) CM3D stiffness. C) Graph representing cellular (+ cells) CM3D stiffness. D) Graph representing acellular and cellular CM3D stiffness at hour 36. To determine the statistical significance between acellular and cellular CM3D respectively, the one-way ANOVA/Kruskall–Wallis test was performed followed by a Dunn’s Multiple Comparison post hoc test. The student’s t-test was performed to determine the statistical significance between acellular and cellular CM3D stiffness at hour 36, a student’s t-test was performed. Values indicate average stiffness or modulus values, and ±SD of n = 4 repeats.

We chose to employ an unconfined uniaxial compression of the whole construct for these determinations because we found that this approach measures the global stiffness properties of a microenvironment with and without embedded cells. It is a recognized technique for characterizing soft cellular tissues [58,59] and cell-material constructs [60]. Each sample’s modulus of elasticity was determined from the slope of linear approximation curve (Fig 5A) composed of the first 10–12% strain values using Eqs 3 and 4. This data measures Young’s modulus, CM3D stiffness, and were selected because the data points generated linear correlations of +/-0.995 [45]. Additionally, we found that maintaining a consistent aspect ratio, *S* (diameter/gauge length of sample) [61], for each CM3D is critical to assure data accuracy. Therefore, test CM3D were all prepared as follows: gauge length of sample = 5.57 *mm*, diameter of sample = 4.78 *mm*. These measurements were employed to calculate the test CM3D area (17.95 *mm*^2^) required for use in the Young’s modulus equation.

Our results show that matrix stiffness, in general, increases in a time dependent manner for both cellular and acellular CM3D cultures (Fig 5B and 5C, respectively). We found that stiffness measurements demonstrated by acellular CM3D only showed a significant increase at (***p ≤ 0.001) between hour 3 (2.35kPa) and hour 6 (7.48kPa) and then plateaus in the remaining time points (ns > 0.05). In cellular CM3D cultures, we also found a gradual increase in stiffness between hour 1 (1.54kPa) and hour 30 (9.72kPa). However, we found a significant increase (*p ≤ 0.05) in stiffness between hour 30 and hour 36 (17.7kPa). Finally, when we compared stiffness outcomes between the two conditions, we observed that cellular CM3D cultures were significantly stiffer (*p ≤ 0.05) in comparison to the acellular CM3D at hour 36 (Fig 5D). This data indicate that cells are interacting with the CM3D matrix from within, resulting in changes in stiffness.

### CM3D Migration Assay Capability

**Fig 6:**
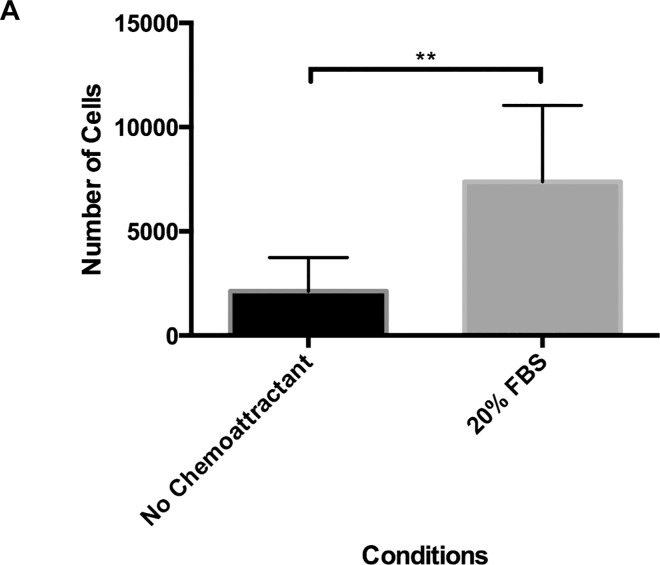
Migration Analysis of CM3D. A) Bar graph represents the number of cells migrating out of CM3D cultures in the presence and absence of a chemoattractant. Values generated by the student’s t-test analysis indicate that average migration and ±SD of n = 8 repeats.

Migration is an essential cell function that give rise to embryonic development, cell response, metastasis and angiogenesis [62]. Current migration assays do not address migration in 3D and they are simplified such that they do not address the complexity of tissues. Migration through CM3D provides a model for cell motility through a tissue-like environment under the influence of a chemoattractant. We were able to demonstrate that cells embedded in CM3D were able to migrate out of the 50mm^3^ CM3D culture into the well when presented with an external chemoattractant. In the absence of a chemoattractant, an average of over 2000 cells were found outside of the CM3D culture. In contrast, when presented with a chemoattractant (20% FBS), a significantly (**p ≤ 0.01) larger number of cells (average of 7000 cells) were found to have migrated out of the CM3D culture (Fig 6). Therefore, we believe CM3D also makes an excellent platform for studying migratory cell behavior in a 3D, tissue-like environment.

## Discussion & Conclusion

In a previous study, we demonstrated that CM3D is an excellent platform for mesenchymal stem cell differentiation [36]. We have since learned that CM3D possesses biomimetic and unique mechanical properties that are appropriate for 3D cell culture. CM3D takes the study of cancer to another level by providing cells a natural environment with FDA approved, nontoxic components, CT and HA. Furthermore, the CM3D construct is formed without the use of potentially hazardous cross-linking agents or methods [8–11]. This eliminates the guesswork in whether additional components from the matrix, such as growth factor enhancement or chemical additives, may be contributing to the host cell’s development.

The hallmarks of a 3D culture platform has been long established by Bissel, et al, using normal and malignantly transformed breast cells [2,63] to show that cellular behavior, morphology, and phenotypes are strongly influenced by the surrounding matrix. In this study, we show that the above-mentioned properties of cells cultured in CM3D contrasted from those of cells cultured on 2D platforms.

Cell viability and proliferation assay in CM3D cultures showed that most viable and proliferative cells reside at the periphery of the construct. This occurrence is due to the presence of a(n) oxygen-, growth factor-, pH- and nutrient- gradient between the periphery and within the matrix [64]. This feature of CM3D may facilitate the development of tumor microenvironments with hypoxic regions, where potent cancer stem cells (CSCs) thrive. The creation of these CM3D-based tumor models could have significant value as there are no known treatments against CSCs [65–67], the main reason for cancer relapse. The ability of 3D culture platforms to support spheroid formation is an important feature since it is widely used in drug screening platforms [68]. After 21 days of culture, we began to notice unique cell staining patterns, where live cells (green) appear more organized and tightly packed in circles toward the periphery of the CM3D culture (Fig 1A). This pattern of viability and proliferation is similar to cell divisions occurring within spheroids [69,70] and strongly implies that CM3D is capable of generating spheroids; however, further investigation is warranted.

The morphology forms that cells take on are dependent on their microenvironment so it is important to understand the regulatory characteristics of the biomaterials used in CM3D [71]. Using SEM, we can study the physiological relevancy of CM3D as a natural ECM. We highlight the addition of a vertical dimension by demonstrating aggregate formation. Furthermore, we found that cells were able to interact with the matrix and each other in many more conformations due to the increase in surface area. In turn, having a wider range of spatial interactions will allow cells to have different downstream effects on their functional ability, response toward various stimuli, and changes on protein and gene expression [72].

Immunofluorescence imaging is known to be a challenge for many 3D culture platforms due to multiple reasons. In certain 3D culture platforms, cells must to be isolated prior to staining by decomposing the matrix; this process can lead to changes in cell morphologies and does not allow the investigator to image the cells in their native 3D structure and their interaction with the microenvironment. In our study, however, we found that the immunostaining of CM3D did not require decomposition of the matrix and enabled us to image the cells without disrupting their native 3D structure (Fig 4). Furthermore, we were able to image cells interacting with their 3D microenvironment in all planes (Fig 4B); therefore, CM3D adds another dimension to imaging. Further demonstrating the versatility of CM3D, we showed that it can be enzymatically decomposed by chitosanase and hyaluronidase enzymes to retrieve the individual cells for flow cytometry. We also demonstrated a functional migration assay where cells migrate out of CM3D in the presence of a chemoattractant.

Our initial findings, produced on the Electroforce 5500 instrument (Fig 5), revealed that acellular CM3D cultures gradually increased in stiffness in a time-dependent manner over 36 hours of incubation. This finding comports well with SEM images showing embedded cells, shrouded in hyaluronan fabrics, “cross-linking” adjacent shards of chitosan (Fig 4). Interestingly, however, when cells are added to CM3D, the stiffness significantly increases after 36hours of culture. This observation can be partially explained by extending our immunofluorescence and SEM results (Fig 4C and 4D). These images show cells forming bridges and attaching themselves to the matrix; as a result, they exert forces on to the matrix. With millions of cells in CM3D, the total cumulative forces exerted from within CM3D will likely increase the stiffness. Therefore, stiffness of CM3D cannot be expressed by a single numerical value. Rather, it displays a stiffness profile the control of which will be the focus of subsequent investigation.

Studies have shown the importance of understanding mechanical properties of the ECM since stiffness and dimensionality have been known to influence cell migration [73]. Cells generate their own mechanical forces by deforming and moving through the ECM network that surrounds them [74,75]. As we gain deeper insight into the properties of CM3D, we expect we will be able to modulate its stiffness such that it correlates with various tissues of the human body. This could give us a highly tunable 3D–cell culture platform to help in the understanding of multi-directional forces exerted on cultured cells and their control of migratory cell behaviors.

*In toto*, these experiments only scratch the surface of the potential of experiments that can benefit from the use of CM3D. Learning from these experiments, we envision various applications, such as models to study tumor infiltration with immune cells, cellular motility in 3D, hypoxia models, etc. Furthermore, the biocompatible nature of CM3D paves the way for future clinical applications such as a translatable drug delivery system.

## Supporting Information

S1 Fig PEC fibers that form during CM3D fabrication. A) Image of HA and CT dry blend taken with a compound microscope (Orig. Mag. 200X). In a non-hydrated mix of the two components, HA particles are electrostatically attached to the shard of CT (orange arrows). The HA particle can also be electrostatically attached to the opposite side of CT shard (red arrow). B) A scanning electron micrograph of lyophilized CM3D construct without cells demonstrating internal positioning of HA-CT PEC fibers, as well as regions of unreacted HA and CT (Orig. Mag. 500X). The grey scale bar for this image is 50μm in length.
(TIF)

S2 Fig Schematic drawing of CM3D set-up. Cells are embedded into CM3D in 5 minutes by PEC following these five simple steps. No UV or crosslinking agents were employed in this process. The final process of extruding the gel can be used in-vitro or in-vivo (as an injectable).
(TIF)

S3 Fig Schematic of the cell migration assay protocol. An outline of the component change and steps required to perform a 4-day migration assay with CM3D. Plates were changed every day with the appropriate media additives (serum starve/chemoattractant) listed above.
(TIF)

S4 Fig SEM images of live and dead cells in CM3D. A) Live cells attached to CM3D and coated with HA particulates (Orig. Mag. 1000X). B) Dead cells resting within CM3D (Orig. Mag. 1500X). The white scale bar for all images is 20μm in length.
(TIF)

S1 Table Summary of stiffness values for acellular and cellular CM3D. The tables summarize stress vs strain measurement of CM3D performed with the unconfined uniaxial compression method. Comparisons were made between acellular vs cellular CM3D stiffness over time. Two experiments with two replicates each were performed to generate the following values.
(DOCX)
