# Supplementary figures and images for: 3D Cell Culture in a Self-Assembled Nanofiber Environment
Source: PLoS One. 2016 Sep 15;11(9):e0162853. doi: 10.1371/journal.pone.0162853 (PMC5025053; doi:10.1371/journal.pone.0162853)

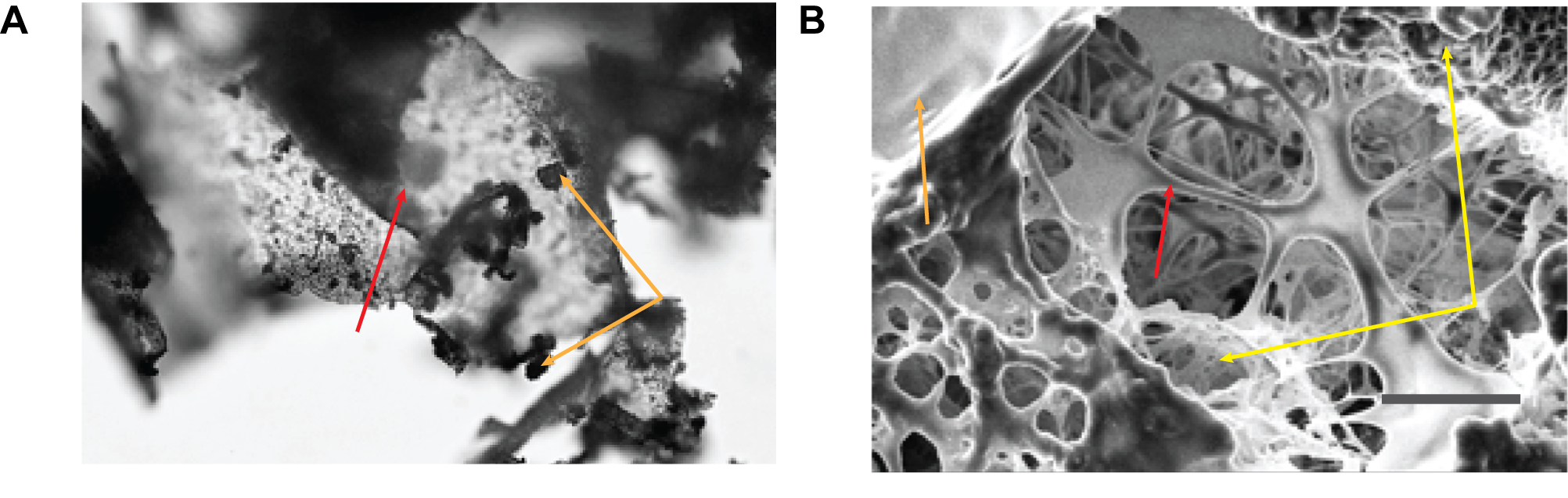

Supplement: S1 Fig — A) Image of HA and CT dry blend taken with a compound microscope (Orig. Mag. 200X). In a non-hydrated mix of the two components, HA particles are electrostatically attached to the shard of CT (orange arrows). The HA particle can also be electrostatically attached to the opposite side of CT shard (red arrow). B) A scanning electron micrograph of lyophilized CM3D construct without cells demonstrating internal positioning of HA-CT PEC fibers, as well as regions of unreacted HA and CT (Orig. Mag. 500X). The grey scale bar for this image is 50μm in length. (TIF) [file pone.0162853.s001.tif]

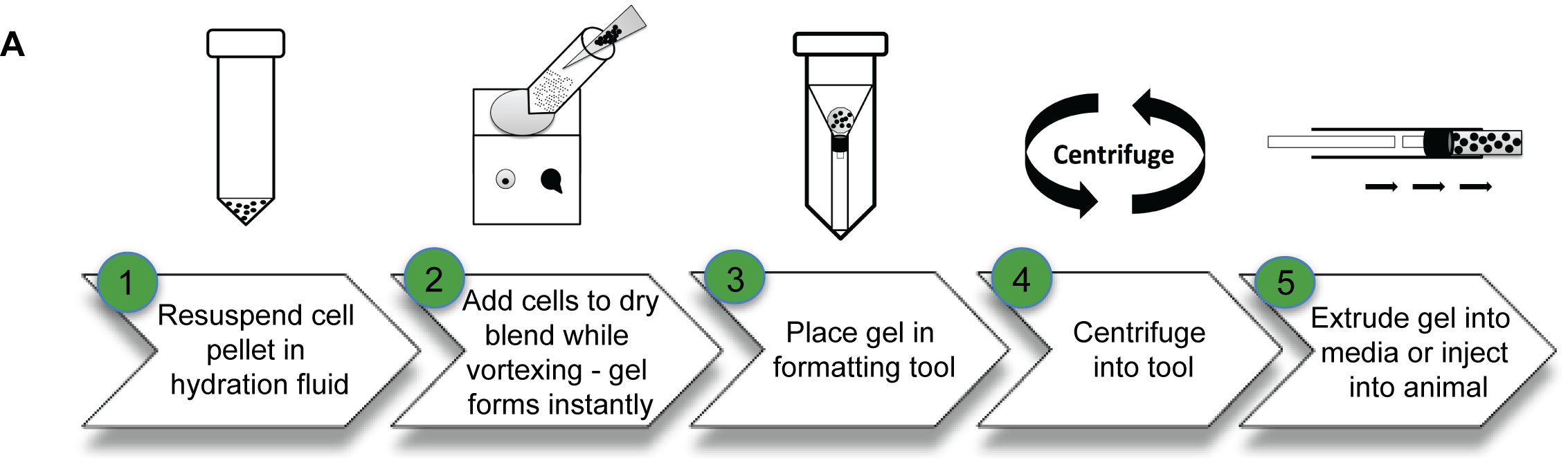

Supplement: S2 Fig — Cells are embedded into CM3D in 5 minutes by PEC following these five simple steps. No UV or crosslinking agents were employed in this process. The final process of extruding the gel can be used in-vitro or in-vivo (as an injectable). (TIF) [file pone.0162853.s002.tif]

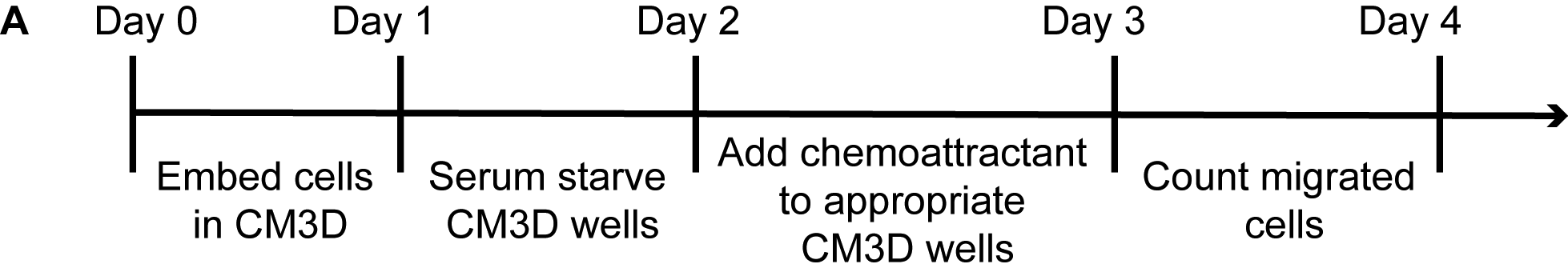

Supplement: S3 Fig — An outline of the component change and steps required to perform a 4-day migration assay with CM3D. Plates were changed every day with the appropriate media additives (serum starve/chemoattractant) listed above. (TIF) [file pone.0162853.s003.tif]

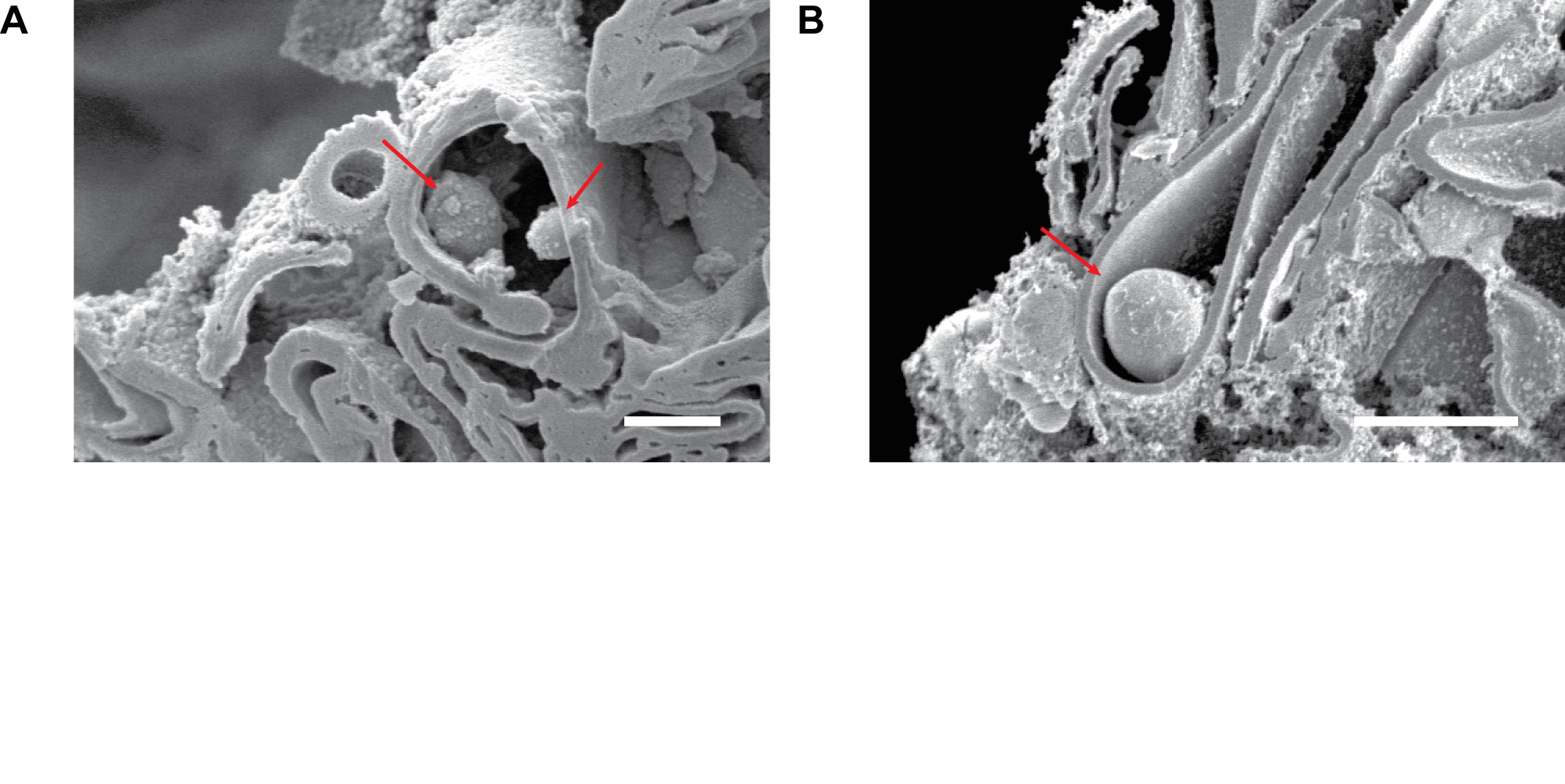

Supplement: S4 Fig — A) Live cells attached to CM3D and coated with HA particulates (Orig. Mag. 1000X). B) Dead cells resting within CM3D (Orig. Mag. 1500X). The white scale bar for all images is 20μm in length. (TIF) [file pone.0162853.s004.tif]
