# Supplementary material for: 3D Cell Culture in a Self-Assembled Nanofiber Environment
Source: PLoS One. 2016 Sep 15;11(9):e0162853. doi: 10.1371/journal.pone.0162853 (PMC5025053; doi:10.1371/journal.pone.0162853)
Supplement: S1 Table — The tables summarize stress vs strain measurement of CM3D performed with the unconfined uniaxial compression method. Comparisons were made between acellular vs cellular CM3D stiffness over time. Two experiments with two replicates each were performed to generate the following values. (DOCX) [file pone.0162853.s005.docx]

**S1 Table.**

| **Time** | **Acellular CM3D Elastic Modulus (kPA)** | | | |
| --- | --- | --- | --- | --- |
|  | **Experiment 1** | | **Experiment 2** | |
|  | **Replicate 1** | **Replicate 2** | **Replicate 1** | **Replicate 2** |
| **Hour 1** | 1.553 | 1.556 | 1.553 | 1.571 |
| **Hour 3** | 2.153 | 2.156 | 2.745 | - |
| **Hour 6** | 8.011 | 7.415 | 8.736 | 5.759 |
| **Hour 12** | 4.947 | 6.756 | 8.411 | 6.678 |
| **Hour 24** | 5.854 | 4.621 | 7.902 | 7.534 |
| **Hour 30** | 6.095 | 5.148 | 7.708 | 7.51 |
| **Hour 36** | 6.615 | 6.908 | 9.253 | - |

| **Time** | **Cellular CM3D Elastic Modulus (kPA)** | | | |
| --- | --- | --- | --- | --- |
|  | **Experiment 1** | | **Experiment 2** | |
|  | **Replicate 1** | **Replicate 2** | **Replicate 1** | **Replicate 2** |
| **Hour 1** | 2.039 | 1.356 | 1.553 | 1.213 |
| **Hour 3** | 3.281 | 5.095 | - | - |
| **Hour 6** | 9.723 | 4.902 | 4.516 | 5.665 |
| **Hour 12** | 3.607 | 5.769 | 8.93 | 6.678 |
| **Hour 24** | 5.941 | 5.16 | 5.452 | 11.317 |
| **Hour 30** | 10.194 | 13.047 | 9.506 | 6.146 |
| **Hour 36** | 12.867 | 25.285 | 14.841 | - |
